# Non-Clinical Studies for Evaluation of 8-*C*-Rhamnosyl Apigenin Purified from *Peperomia obtusifolia* against Acute Edema

**DOI:** 10.3390/ijms18091972

**Published:** 2017-09-14

**Authors:** Cinthia I. Tamayose, Paulete Romoff, Daniela O. Toyama, Henrique H. Gaeta, Caroline R. C. Costa, Mariana N. Belchor, Bruna D. Ortolan, Leosvaldo S. M. Velozo, Maria A. C. Kaplan, Marcelo J. P. Ferreira, Marcos H. Toyama

**Affiliations:** 1 Curso de Química, Universidade Presbiteriana Mackenzie, Rua da Consolação, São Paulo 01302-907, Brazil; cinthiatamay@gmail.com (C.I.T.); romoff@mackenzie.com.br (P.R.); 2 Faculdade de Odontologia, Universidade Camilo Castelo Branco (UNICASTELO), São Paulo 15600-000, Brazil; gaveira@yahoo.com.br; 3 Universidade Estadual Paulista (UNESP), Campus do Litoral Paulista, Praça Infante Dom Henrique s/n Bairro: Parque Bitaru; São Vicente, CEP 11330-900, Brazil; henriquehg@gmail.com (H.H.G.); carolsbert@gmail.com (C.R.C.C.); mary_novo@hotmail.com (M.N.B.); bruna-ortolan@hotmail.com (B.D.O.); 4 Instituto de Biologia—Universidade do Estado do Rio de Janeiro (UERJ), Rio de Janeiro 20550-900, Brazil; velozo72@hotmail.com (L.S.M.V.); imkaplan@uol.com.br (M.A.C.K.); 5 Departamento de Botância, Instituto de Biociências, University of São Paulo, São Paulo 05508-900, Brazil; marcelo-pena@uol.com.br

**Keywords:** *Peperomia obtusifolia*, Piperaceae, flavonoid, snake venom phospholipase A2, myonecrosis, edema

## Abstract

Compound 8-*C*-rhamnosyl apigenin (8CR) induced a moderate reduction in the enzymatic activity of secretory phospholipase A2 (sPLA2) from *Crotalus durissus terrificus* and cytosolic phospholipase A2 (cPLA2), but the compound also significantly inhibited the enzymatic activity of the enzyme cyclooxygenase. In vitro assays showed that the compound induced a slight change in the secondary structure of sPLA2 from *Crotalus durissus terrificus* snake venom. In vivo assays were divided into two steps. In the first step, the 8CR compound was administered by intraperitoneal injections 30 min prior to administration of sPLA2. In this condition, 8CR inhibited edema and myonecrosis induced by the sPLA2 activity of *Crotalus durissus terrificus* in a dose-dependent manner by decreasing interleukin-1β (IL-1β), tumor necrosis factor α (TNF-α), prostaglandin E2 (PGE2), and lipid peroxidation. This has been demonstrated by monitoring the levels of malondialdehyde (MDA) in rat paws after the course of edema induced by sPLA2. These results, for the first time, show that sPLA2 of *Crotalus durissus terrificus* venom induces massive muscle damage, as well as significant edema by mobilization of cyclooxygenase enzymes. Additionally, its pharmacological activity involves increased lipid peroxidation as well as TNF-α and IL-1β production. Previous administration by the peritoneal route has shown that dose-dependent 8CR significantly decreases the enzymatic activity of cyclooxygenase enzymes. This resulted in a decrease of the amount of bioactive lipids involved in inflammation; it also promoted a significant cellular protection against lipid peroxidation. In vivo experiments performed with 8CR at a concentration adjusted to 200 μg (8 mg/kg) of intraperitoneal injection 15 min after sPLA2 injection significantly reduced sPLA2 edema and the myotoxic effect induced by sPLA2 through the decrease in the enzymatic activity of cPLA2, cyclooxygenase, and a massive reduction of lipid peroxidation. These results clearly show that 8CR is a potent anti-inflammatory that inhibits cyclooxygenase-2 (COX-2), and it may modulate the enzymatic activity of sPLA2 and cPLA2. In addition, it was shown that *Crotalus durissus terrificus* sPLA2 increases cell oxidative stress during edema and myonecrosis, and the antioxidant properties of the polyphenolic compound may be significant in mitigating the pharmacological effect induced by sPLA2 and other snake venom toxins.

## 1. Introduction

More recently, plant extracts have been used as alternative anti-venom compounds for the management of snake bites and are used in conjunction with conventional antibody therapies [1,2]. In Brazil, *Crotalus durissus terrificus* venom is responsible for approximately 10% of snake bites and has a high mortality due to the toxic action of crotoxin exhibiting various pharmacological activities, including neurotoxicity, myotoxicity, nephrotoxicity, cardiotoxicity, and inflammation [3,4]. Recent studies have shown that snake venom secretory phospholipase A2 (sPLA2) has a mechanism of action highly like that of human sPLA2s, and the pharmacological activity of sPLA2 involves the generation of arachidonic acid. This may also involve the cross-talk between cytosolic phospholipases A2 (cPLA2) and other enzymes involved in arachidonic acid metabolism and associated with increase of cellular oxidative stress, such as pharmacological events induced by human secretory phospholipase A2 [5,6,7]. Recent studies show that both cyclooxygenase-2 (COX-2) and cytosolic phospholipase A2 (cPLA2), which are rigorously regulated by several mediators in several species, including several transcription factors activated during the inflammatory process, hydrolyze membrane phospholipids, which results in the release of arachidonic acid (AA), which is further converted by COX-2 and prostaglandin (PG) synthases to biologically-active PGs [8]. The genus *Peperomia* is used as an ornamental species, and others species have been used in folk medicine in some countries due to their anti-tumor, anti-inflammatory, antibacterial, and analgesic activities [9], and phytochemical studies in species of *Peperomia* showed the presence of a wide range of natural compounds, including lignans, polyketides, chromenes and chromanes, quinones, and flavonoids [10,11,12]. The 8-*C*-rhamnosyl apigenin (8CR) purified from *Peperomia obtusifolia* is an unpublished compound, and no pharmacological activity has been described for it. What is known about *Peperomia obtusifolia* is that it is a well-known ornamental foliage plant found in Mexico and parts of northern South America, and previous chemical research has demonstrated the presence of various polyphenolic compounds [13,14]. The aim of this study is to investigate the effect of 8-*C*-rhamnosil apigenin isolated from *Peperomia obtusifolia* on the toxic and pharmacological effects induced by purified secreted phospholipase A2 and on COX-2 and cPLA2.

## 2. Results

### 2.1. Structural and Biological Characterization of 8-C-Rhamnosyl Apigenin (8CR)

**Figure 1:**
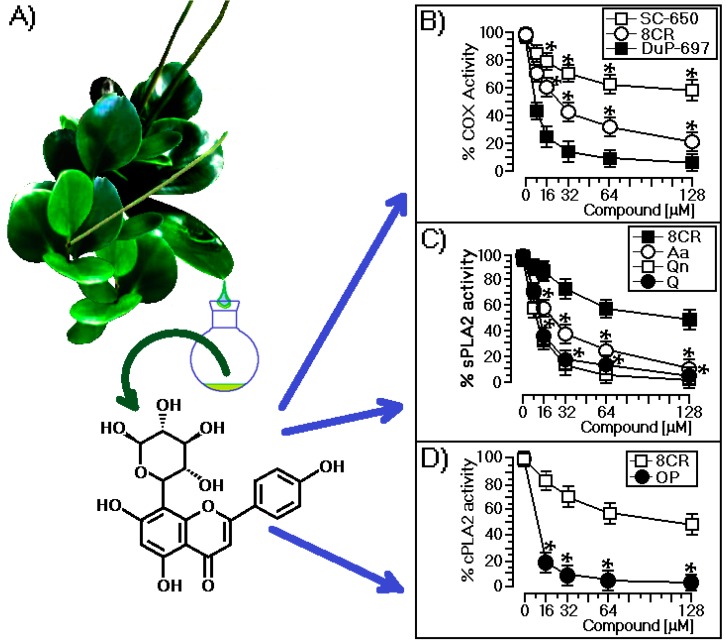
(**A**) Chemical structure of 8-*C*-rhamnosyl apigenin (8CR) isolated from *Peperomia obtusifolia* and the botanical illustration of the plant; (**B**) Comparative effects of 8CR, DuP-697, and SC-560 on cyclooxygenase-1 (COX-1) and COX-2 enzymatic activities. Each point represents the mean ± SEM of six animals in the percentage of remaining enzymatic activity of COX-1 or COX-2 without inhibitors; (**C**) Effects of 8CR, aristolochic acid (Aa), quercetin (Q), and quercitrin (Qn) on the enzymatic activity of sPLA2 isolated from *Crotalus durissus terrificus*; (**D**) The enzymatic effect of COX-2 in the presence of oleyloxyethyl phosphorylcholine (OP) and 8CR. All of the compounds used in this section were dissolved according to the supplier’s information by adjusting the concentrations for 128, 64, 32, and 16 μM, and all assays were done with the material provided by the manufacturer. * indicates significant differences relative to a standard. All analyses were performed using analysis of variance (ANOVA, *p* < 0.05), and each bar represents *n* = 5.

*Peperomia* species have been the source of various bioactive compounds, such as aromatic compounds and polyketides [12,13,14,15,16,17]. Previous studies with *P. obtusifolia* reported the isolation of chromanes, flavonoids, and lignans [18,19]. The n-BuOH phase from the MeOH extract of the aerial parts of *P. obtusifolia* was chromatographed on a Sephadex LH-20 column (Björkgatan 30, 751 84 Uppsala, Sweden), which afforded a phenolic compound characterized by analysis of its spectroscopic data. The UV spectrum revealed characteristic flavone absorptions at 269 and 331 nm. The 1H nuclear magnetic resonance (NMR) spectrum of the compound showed a singlet at δ 6.70 consistent with the H-3 of flavones, and this was supported by the observation of a carbon signal at δ 102.5 associated with the C-3 in its ^13^C NMR spectrum. The B ring of the flavone was oxygenated only at C-4′ due to the two doublet signals at δ 8.10 (2H, d, *J* = 9.0 Hz) and 7.10 (2H, d, *J* = 9.0 Hz) assigned to H-2′/H-6′ and H-3′/H-5′, respectively. This substructure was confirmed through the signals at δ 124.9, 130.4, 115.8, and 164.7 obtained from the ^13^C NMR spectrum and assigned to C-1′, C-2′/C-6′, C-3′/C-5′, and C-4′, respectively. An additional singlet at δ 6.52 bonded to a carbon atom at δ 96.7 suggested a penta-substituted A-ring. This signal exhibited a heteronuclear multiple-bond correlation (HMBC) with C5, C7, and C10, which confirmed the location of the aromatic proton at C6. The presence of two additional doublets at δ 5.04 (1H, d, *J* = 9.8 Hz) and δ 0.62 (3H, d, *J* = 6.0 Hz) bonded to carbons at δ 72.2 and δ 18.2, respectively, suggested that the rhamnosyl moiety was C-attached to the flavone. The sugar linkage was determined to be C–C from the relatively upfield anomeric carbon resonances at δ 72.2, in contrast to the anomeric carbons of *O*-glycosides, which normally resonate at approximately δ 100.0 [20]. Thus, the rhamnose was determined to be bonded directly to the C-8 of the flavone. The sugar position linkage was confirmed by observation of the HMBCs between H-1″ and C-7, C-8, C-9, and C-3″. A comparison of the spectroscopic data with those reported in the literature allowed for the identification of the isolated flavonoid as a 5,7,4′-trihydroxyflavone 8-*C*-rhamnoside (8-*C*-rhamnosyl apigenin; 8CR), which is shown in Figure 1A. In Figure 1A, we also present a botanical illustration—*Peperomia obtusifolia*—to illustrate the plant; the figure was captured from the Internet.

In Figure 1B, we showed that 8CR significantly reduced the enzymatic activity of cyclooxygenase and presented an IC_50_ of 28.6 μM, but its activity was lower when compared to the effect of 5-bromo-2-(4-fluorophenyl)-3-(4-(methylsulfonyl)phenyl)-thiophene (DuP-697), which is a selective COX-2 inhibitor like other COX-2 inhibitors and showed an IC50 7.67 μM. In contrast, the 5-(4-chlorophenyl)-1-(4-methoxyphenyl)-3-(trifluoromethyl)-1H-pyrazole (SC-560) compound showed low inhibitory activity because it is a highly-selective compound for COX-1. In Figure 1C, 8CR induced only a marginal inhibitory effect on the enzymatic activity of sPLA2, exhibiting IC_50_ of 109.4 μM, whereas other flavonoids, such as quercetin (Q) and quercitrin (Qn), showed IC_50_ values of 13.5 and 9.2 μM, respectively. Commercial inhibitor compound sPLA2 (aristolochic acid, Aa) showed an IC_50_ value of 22.4 μM. The results presented in Figure 1C do not allow us to classify it as a specific inhibitor of sPLA2 compared with other flavonoids that were evaluated. In Figure 1D, we show the effects of 8CR and the oleyloxyethyl phosphorylcholine (OP) compound on the enzymatic activity of the cloned human cPLA2. Both enzymes were subjected to the same assay conditions. 8CR showed an IC_50_ value of 134 μM against the enzymatic activity of cPLA2, but this inhibitory effect induced by 8CR was insignificant when compared with the OP inhibitor.

### 2.2. Structural Shifts Induced by 8CR on the sPLA2 Molecule

**Figure 2:**
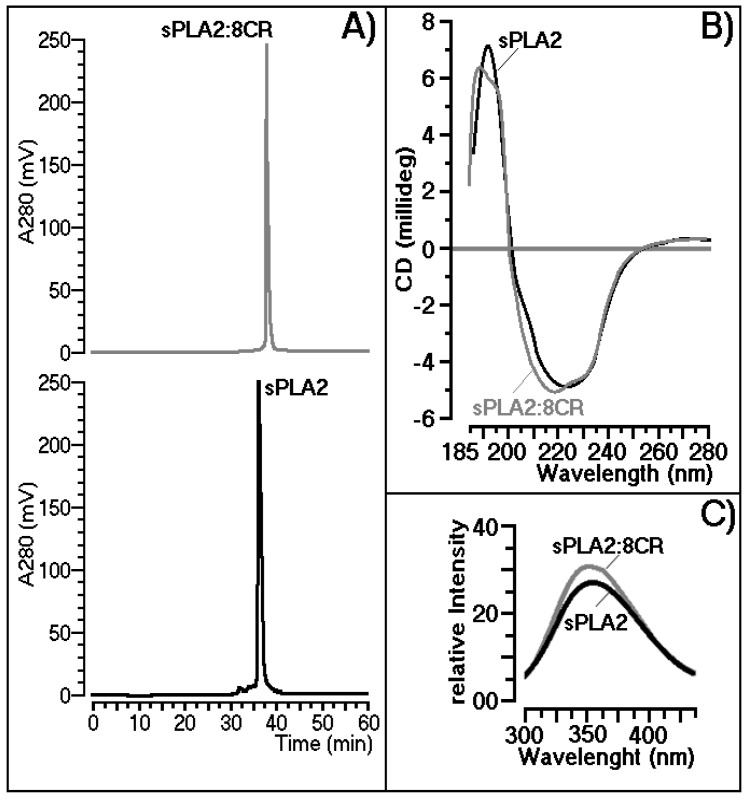
Comparison of the native secretory phospholipase A2 (sPLA2) and sPLA2 treated with 8CR in relation to: (**A**) chromatographic retention times; (**B**) circular dichroism curves and (**C**) fluorescence of the protein.

The incubations of *C. d. terrificus* sPLA2 with purified 8CR (mol/mol) were performed according to the procedure described previously [21]. The results of reverse phase HPLC analysis showed that 8CR could form a stable complex with sPLA2, and that 8CR also changed the molecular structure of sPLA2, since the retention time of sPLA2 changed significantly in the presence of 8CR (Figure 2A). Compound 8CR, when incubated with sPLA2, also induced slight changes in the level of the secondary structure. Both sPLA2 and sPLA2:8CR were dissolved in 10 mmol/L sodium phosphate buffers, pH 7.4, and both proteins had their concentrations adjusted to 8.7 mmol/L. Both proteins were subjected to the same conditions of circular dichroism analysis using a J720 spectropolarimeter (Jasco Corp., Tokyo, Japan). Data collection was performed at room temperature with a scanning speed of 100 nm/min. Nine scans were obtained for each sample, and all spectra were corrected by subtracting buffer blanks (Figure 2B). The relative intrinsic fluorescence intensity of native sPLA2 or 8CR-treated sPLA2 was monitored with a Shimadzu spectrofluorimeter. Reaction mixtures of 2.0 mL in a 1-cm path length quartz cuvette consisting of 100 mmol/L Tris–HCl buffer (pH 7.4), sPLA2 (200 μg/mL), and 5 mmol/L calcium. Fluorescence was measured between 300 and 450 nm after excitation at 280 nm (Figure 2C).

### 2.3. Protective Effect of 8CR against the Pharmacological Effects of sPLA2

**Figure 3:**
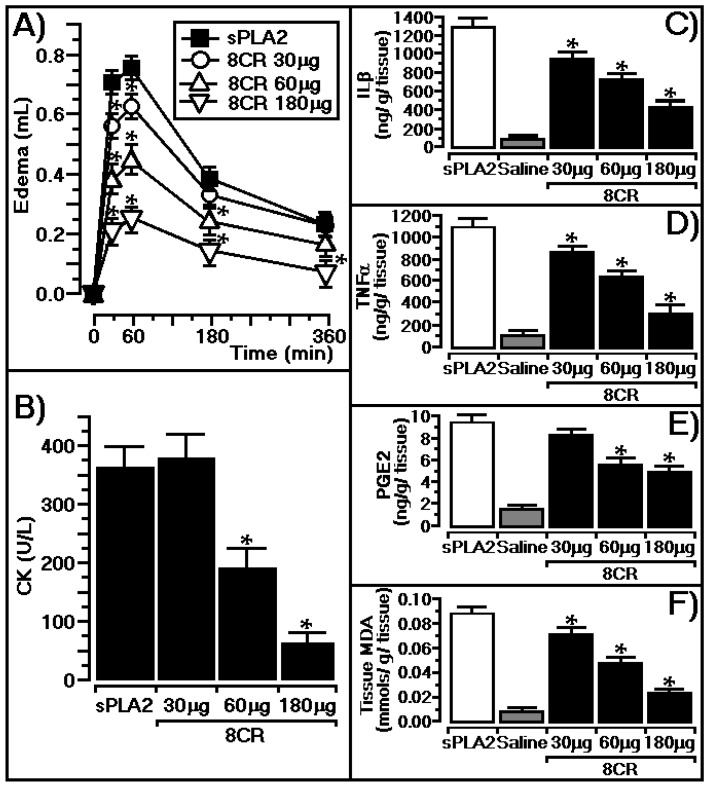
(**A**) Acute edema volumes at different times and administration doses of 8CR; (**B**) the protective effect of the flavonoid on the myotoxic activity induced by sPLA2; and in (**C**–**F**), all animals were treated with 30 μg (1.2 mg/kg), 60 μg (2.4 mg/kg), and 180 μg (7.2 mg/kg) 8CR prior to injection of sPLA2. After experiments, tissue homogenate from each animal was prepared for specific analysis following the procedures described in each protocol supplied by the manufacturers. * indicates significant differences relative to a standard. All analyses were performed using analysis of variance (ANOVA, *p* < 0.05), and each bar represents *n* = 5.

The results presented in Figure 3A indicate that the edema induced from sPLA2 isolated from *Crotalus durissus terrificus* was strongly reduced in a dose-dependent manner by three different concentrations of 8CR. Acute edema induced by sPLA2 at times of 30 and 60 min were the most affected by a previous intraperitoneal (i.p.) injection of the compound. The sPLA2 isolated from *Crotalus durissus terrificus* showed maximum edema at 30 to 60 min, and exhibited edema values of 0.71 ± 0.018 and 0.77 ± 0.015 mL, respectively. Figure 3A presents edema values of 0.56 ± 0.021 and 0.62 ± 0.012 mL (*n* = 5) for animals that received 30 μg of i.p. doses of 8CR (1.2 mg/kg) at 30 and 60 min, respectively. Moreover, for animals that received 60 μg of i.p. doses of 8CR (2.4 mg/kg), the edema values at 30 and 60 min were 0.37 ± 0.023 and 0.45 ± 0.01 mL (*n* = 5, * *p* < 0.05), respectively. In addition, Figure 3A indicates that 180 μg of an i.p. dose of flavonoid (7.2 mg/kg) injected into the animals before the administration of sPLA2 exhibited edema values of 0.21 ± 0.021 mL (*n* = 5, * *p* < 0.05 at 30 min) and 0.26 ± 0.018 mL (*n* = 5, * *p* < 0.05 at 60 min). Edema induced by sPLA2 in the animals previously treated with indomethacin (positive control, 10 mg/kg) diminish edema values of 0.25 ± 0.018 mL (*n* = 5, * *p* < 0.05) at 30 min and 0.32 ± 0.022 mL (*n* = 5, * *p* < 0.05) at 60 min, respectively.

Figure 3B presents the protective effect of the flavonoid against myotoxic activity induced by sPLA2. The native phospholipase A2 induced an increase in the plasma creatine kinase (CK) values of 368 ± 63 (*n* = 6), whereas the administration of 8CR at concentrations of 60 μg (2.4 mg/kg) and 180 μg (7.2 mg/kg) effectively decreased the myonecrosis activity induced by the native sPLA2 (CK values of 182 ± 34 and 52 ± 16 U/L, respectively; *n* = 5, * *p* ≤ 0.05; Figure 3B). Indomethacin (10 mg/kg) significantly diminish myonecrosis induced by sPLA2 at 325 ± 23 U/L, respectively; *n* = 5, * *p* ≤ 0.05. In addition, we observed that sPLA2 also induced a significant mobilization of interleukin-1β (IL-1β), tumor necrosis factor α (TNFα), and prostaglandin E2 (PGE-2), and significantly increased lipid peroxidation. IL-1β is recognized as a key mediator of inflammation, is an inducible cytokine, and is not generally expressed in healthy cells or tissue. TNFα is a powerful pro-inflammatory agent that regulates many facets of macrophage function. In particular, some studies demonstrated that PGE-2 is an important prostaglandin produced by acute inflammation and enhanced lipid peroxidation, which occurs during oxidative stress and results in the generation of lipid peroxidation end products, such as malondialdehyde (MDA). The results presented in Figure 3C–F show that the previous administration of 8CR in a dose-dependent manner strongly decreases IL-1β, TNFα, PGE-2, and lipid peroxidation, and better results were observed for 8CR concentrations adjusted at 180 μg (7.2 mg/kg) for each animal. Thus, i.p. application of 8CR in different doses induced a dose-response decrease in IL-1β, TNFα, PGE-2, and tissue MDA levels in the paws (*n* = 5, *p* < 0.05).

### 2.4. Antiophidian Activity Effect of 8CR against the Pharmacological Effects of sPLA2

**Figure 4:**
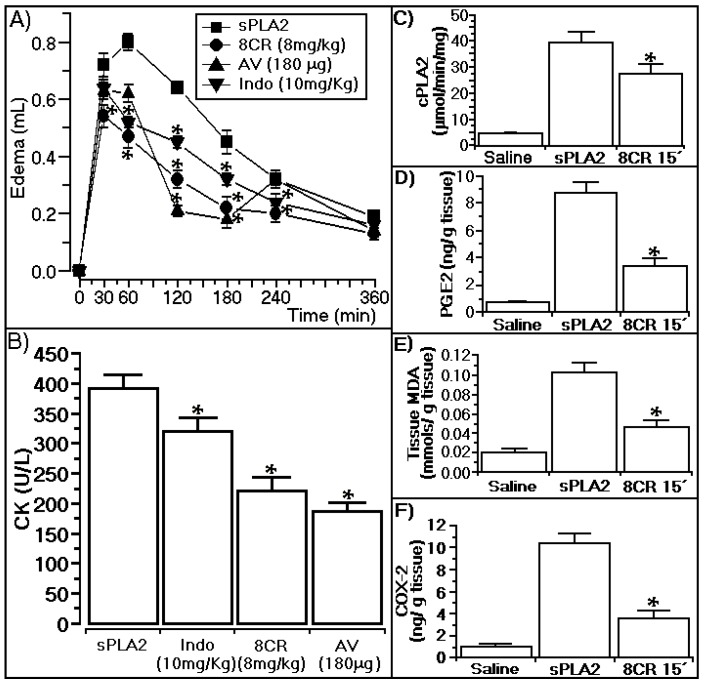
Effects of 8-*C*-rhamnosyl apigenin injected 15 min after the injection of sPLA2 from *Crotalus durissus terrificus*. In (**A**) we observe the anti-inflammatory effect of 8CR that neutralizes the edema induced by sPLA2; In (**B**), we see the protective effect of the flavonoid on the myotoxic activity induced by sPLA2; (**C**–**F**) show the hind paw tissue homogenate quantification of cPLA2, PGE-2, MDA, and COX-2. All biochemical determinations were established by swollen hind paw tissue homogenate analysis of five animals. In (**C**–**E**), each column of biochemical determination represents the mean ± SEM of five animals and * statistically significant differences (*n* = 5, *p* < 0.05). All animals were treated with 200 μg (8 mg/kg) 8CR prior to injection of sPLA2, and after the experiments swollen hind paw tissue homogenate from each animal was prepared for specific analysis following the procedures described in each protocol supplied by the manufacturers. AV (crotalic and bothropic commercial veterinary propose antivenom, Lema-Injex, São Paulo, Brasil). Indo (Indomethacin, Sigma-Aldrich, St. Louis, MO, USA).

The results presented in Figure 4A show that 8CR (8 mg/kg) administered 15 min after injection of sPLA2 reduced edema induced by sPLA2 at 60, 180, and 360 min from 0.81 ± 0.012, 0.47 ± 002 and 0.22 ± 0.01 mL to 0.47 ± 0.021, 0.23 ± 0.018 and 0.21 ± 0.01 mL, respectively (*n* = 5, * *p* ≤ 0.05). 8CR’s inhibition of edema was more effective than the anti-venom principally after 30 and 60 min, and indomethacin at all times observed. Figure 4B shows that the administration of 8CR at concentrations of 200 μg (8 mg/kg) decreased the myonecrosis induced by the native sPLA2 (CK values of 0.396 ± 27 U/L) to 0.238 ± 32 U/L (*n* = 5, * *p* ≤ 0.05; Figure 4B). 

As shown in Figure 4B, indomethacin (the positive control for edema) showed a marginal effect against myonecrosis induced by native sPLA2, and commercial snake anti-venom did not show this difference with 8CR. After three hours post-injection of sPLA2 and inoculation of 8CR, a group of five animals were sacrificed and swollen hind paw tissue homogenate was subjected to biochemical analysis for determination of COX-2, MDA, PGE-2, and cPLA2 analysis. These biochemical analyses showed that edema and myonecrosis induced by sPLA2 from *Crotalus durissus terrificus* involve prostaglandin overproduction, such as PGE-2, and an increase of oxidative stress. The results presented in Figure 4C–F show that the administration of 8CR 15 min after injection of sPLA2 strongly decreases PGE-2, lipid peroxidation, and COX-2, and significantly inhibited enzymatic activity of cPLA2.

## 3. Discussion

### 3.1. Structural and Biological Characterization of 8CR

No previous study has described the importance of COX-2 enzymatic activity in the action of sPLA2 from the venom *Crotalus durissus terrificus*. Studies performed by Moreira et al. [22] using animals after intraperitoneal injection of *Bothrops asper* sPLA2 and *Crotalus durissus terrificus* demonstrated that only *Bothrops asper* sPLA2 induced an increase in cytosolic expression, and COX-2 levels by leukocytes. Our results using sPLA2 from *Crotalus durissus terrificus* demonstrated the role and importance of COX-2 during the edema and myonecrosis induced by this protein. In addition, the pharmacological activities of sPLA2 were strongly dose-dependently reduced by 8-C-rhamnosyl apigenin by reducing enzymatic activity and COX-2 expression. Consequently, the ability of COX-2 to synthesize prostaglandins has been shown to be an important factor for edematogenic and myotoxic activity. The results of 8CR treatments demonstrated cross-talk between *Crotalus durissus terrificus* and COX-2 sPLA2 in both pharmacological activities in edema and myonecrosis. The results presented here showed that 8CR inhibits the COX-2 enzyme more specifically than sPLA2 or cPLA2, but these results did not rule out the possibility that the higher concentrations of 8CR inhibited the enzymatic systems cPLA2 and sPLA2. Thus, 8CR appears to be a good candidate for the development of a new class of drugs that moderately reduces the enzymatic activity of two key molecules, sPLA2 and cPLA2, which may decrease the production of arachidonic acid. In addition, 8CR significantly decreases the production of bioactive prostanoids by COX-2 inhibition.

### 3.2. Structural Shifts Induced by 8CR on the sPLA2 Molecule

Drugs fail in the clinic for two main reasons: the first is that they do not work, and the second is that they are not safe and a great amount of money was involved. Thus, the pharmaceutical industry and, more recently, some academic centers, have streamlined a number of early processes to identify molecules that possess suitable characteristics to make acceptable drugs, including choice and specific analysis against molecular targets, such as protein [23,24]. In recent years, our groups have focused several efforts to use sPLA2 from *Crotalus durissus terrificus* as a molecular target for the evaluation of the potential application of natural products against acute edema. These investigations involve spectroscopic, chromatographic, enzymatic, and other studies for monitoring the effects of these compounds on the structure and function of sPLA2. Our investigation showed that, in the case of sPLA2 from *Crotalus durissus terrificus* venom, 8CR did not induce significant structural modification, and in the case of sPLA2, the smaller shifts observed did not affect the structure of sPLA2.

### 3.3. In Vivo Protective Effect of 8CR against the Pharmacological Effects of sPLA2

In Figure 3, all the results clearly show that edema and myonecrosis induced by sPLA2 involve the mobilization of several pro-inflammatory molecules, an increase in the enzymatic activity of cyclooxygenase by stimulation of COX-2 expression, and oxidative stress, which appear to be important elements for sPLA2-induced cellular damage. PGE-2 is one of several products generated by the overexpression of COX2, and has been widely characterized as an important eicosanoid involved in many inflammatory conditions, and may stimulate the generation of hydroxyl radicals and increase lipid peroxidation and cellular apoptosis [25,26]. The quantification of MDA was used to estimate the oxidative damage of cells that could lead to an oxidative attack on polyunsaturated lipids. During acute inflammation, tumor necrosis factor α (TNF-α) and IL-1β are responsible for a wide range of signaling events in cells, which lead to necrosis or apoptosis by increasing cellular oxidative stress [27,28,29]. Our results clearly showed that i.p. previous to 8CR significantly decreased the mobilization of pro-inflammatory cytokines induced by sPLA2, and significantly decreased the levels of MDA in a dose-dependent manner. The results also suggest that 8CR can inhibit lipid peroxidation and, consequently, the cytotoxic effects induced by sPLA2 from snake venom by decreasing free radical levels in cells and the COX-2 metabolism should be involved in oxidative stress [30].

### 3.4. Anti-Ophidian Effect of 8CR against the Pharmacological Effects of sPLA2

The results presented in Figure 4 show that 8CR administered intraperitoneally 15 min after sPLA2 injection of *Crotalus durissus terrificus* was able to abolish edema, as well as the myotoxic effect resulting from administration of the toxin. This protective or antiophidic effect of compounds at the dose of 200 μg (8 mg/kg) per animal virtually abolished the COX-2 enzyme activity; this inhibition probably should have induced a lower production of PGE-2, and probably of other pro-inflammatory interleukins. In addition, 8CR was also effective in neutralizing lipid peroxidation, which was indirectly measured by the biochemical quantification of MDA from swollen hind paw tissue homogenate aliquots. On the other hand, these results were obtained with the compound being administered at a dose of 200 μg (8 mg/kg) of 8CR, which also inhibited the activity of cPLA2. Thus, 8CR neutralized the effect of *Crotalus durissus terrificus* sPLA2 by inhibiting the major enzymes involved in arachidonic acid metabolism and by the ability to neutralize the toxic effects of lipid peroxidation. In addition, our results show that 8CR showed much better therapeutic qualities than commercial anti-venom, mainly in the completion of edema or myotoxic activity induced by *Crotalus durissus terrificus* sPLA2.

## 4. Materials and Methods

### 4.1. Materials and General Experimental Procedures

*Crotalus durissus terrificus* whole dried venom was purchased from Bio-Agents serpentary (Faz Boa Esperanca, S/N, Zona Rural, Batatais, SP, CEP 14300-000, Brazil). The solvents, chemicals, and reagents used for protein purification and characterization (high-performance liquid chromatography (HPLC)-grade or higher) were acquired from Sigma-Aldrich Chemicals (St. Louis, MO, USA), Merck (Kenilworth, NJ, USA), and Bio-Rad (Hercules, CA, USA). Male Swiss mice (25 g) were obtained from the Multidisciplinary Center for Biological Research (CEMIB) of the State University of Campinas (UNICAMP, São Paulo, Brazil). The animals were maintained under standard conditions (22 ± 2 °C; 12 h light/dark cycle) with food and water available ad libitum. All animal experiments were performed in accordance with Brazilian Laws for the Care and Use of Laboratory Animals, and the protocols were approved by the Protocol No. 014-CEUA (23 August 2016) and Protocol No. 019-CEUA (23 August 2016). Briefly, 1H and 13C NMR spectra were recorded at 300 and 75 MHz, respectively, on a Bruker (Billerica, MA, USA) DPX-300 spectrometer. HMBCs were recorded on a Bruker Avance DRX-500 spectrometer. CD3OD (Tédia, Aparecida de Goiânia, Brazil) was used as the solvent, and TMS (Sigma-Aldrich) was used as the internal standard. Chemical shifts are reported in δ (units: ppm) and coupling constants (*J*) in Hz.

### 4.2. Extraction and Purification of 8CR

Aerial parts of *Peperomia obtusifolia* (L.) A. Dietr. (Piperaceae) were collected in Petrópolis, Rio de Janeiro, RJ, in February 2008. The identification of the plant was performed by Prof. Elsie Franklin Guimarães, Botanical Garden of Rio de Janeiro. A voucher specimen (RB: 393491) was deposited in the Herbarium of Rio de Janeiro Botanical Garden, Rio de Janeiro, Brazil. Fresh aerial parts (1000 g) were cut and extracted with methanol (MeOH) at room temperature. The crude methanolic extract (18.58 g) was suspended in MeOH:H_2_O (1:2 *v*/*v*) and successively partitioned into hexane, CH_2_Cl_2_, EtOAc, and n-BuOH. Part of the n-BuOH phase (325 mg) was subjected to column chromatography (CC) over a Sephadex LH-20 column, and eluted with MeOH to yield seven groups (N1–N7). The N4 fraction (43.0 mg), which is composed of a pure compound, was identified through 1H and 13C nuclear magnetic resonance (NMR) spectroscopy as a rhamnosyl apigenin derivative. The correct position of the sugar was assigned by HMBCs, and the compound was characterized as 8-*C*-rhamnosyl apigenin (8CR; Figure 1) in accordance with data in the literature [31,32].

### 4.3. Purification of Secretory Phospholipase A2

Fractionation of phospholipase A2 from the total venom of *Crotalus durissus terrificus* was purified by two steps. In the first step, the total venom was injected into a molecular exclusion HPLC column (Superdex 75, 1 × 60 cm, Pharmacia, London, UK), and the chromatographic run was performed with a flow rate of 0.2 mL/min for the elution of crotoxin. After confirming the enzymatic activity and biochemical analyzes, as described in [9], it was subjected to reverse-phase chromatography using a μ-Bondapack C18 column (0.39 × 30 cm) with a flow rate of 1 mL/min for fraction elution of sPLA2 and crotapotin. The fractions of sPLA2 were then submitted to enzymatic assays and analyzed in electrophoresis in sodium dodecyl sulphate-polyacrylamide gel electrophoresis (SDS-PAGE) and mass spectrometry on a matrix-assisted laser desorption ionization time-of-flight (MALDI-TOF) mass spectrometer, as previously described [32].

### 4.4. Cytosolic PLA2, sPLA2, and COX-1 and -2

For the cytosolic PLA2 assay, we used a cPLA2 Assay Kit (catalog No. 765021), purchased from Cayman Chemical (Ann Arbor, MI, USA), and procedures were conducted according to the manufacturer’s instructions. For the test, cytosolic phospholipase A2 (Holzer diagnostika, Hölzel Diagnostika Handels GmbH, Hohenzollernring 38, 50672 Cologne, Germany) was prepared following the manufacturer’s recommendation. Moreover, inhibitor stock solutions of 8CR and oleyloxyethyl phosphorylcholine, a potent inhibitor of PLA2 (Santa Cruz Biotechnology, Dallas, TX, USA), were adjusted to prepare a final concentration of 1 μmol/L that was dissolved in 100 μL of DMSO and added to 10 mL of cPLA2 assay buffer (stock solution). After the preparation of each stock solution, we adjusted the specific volume of each inhibitor concentration, and mixed and stored them at room temperature in amber glass bottles until use. In this work, we performed this assay in sextuplicate, at 0, 16, 32, 64, and 128 μM of 8CR or OP. After preparation of recombinant cPLA2 under the same conditions indicated for the cPLA2 assay kit, we performed the enzymatic assay as described for the cPLA2 assay kit.

For sPLA2 inhibition, sPLA2 activity was measured by following a previously-described protocol [32] for a 96-well plate assay using 4-nitro-3-octanoyloxybenzoic acid (4N3OBA or NOBA, Enzo Life Sciences, Inc. Farmingdale, NY, USA) as the substrate. 8CR and aristolochic acid (Aa), quercetin (Q), and quercetrin (Qn) were adjusted to prepare a final concentration of 1 mmol/mL that was dissolved in 100 μL of DMSO and added to 10 mL of Tris-HCl 50 mmol/L buffer pH 8.0 (stock solution). After the preparation of each stock solution, we adjusted the specific volume of each inhibitor concentration, then mixed and stored them at room temperature in amber glass bottles until use. In this work, we made this evaluation in sextuplicate, at 0, 16, 32, 64, and 128 nmol/L for all compounds, including 8CR, Aa, Q, and Qn. These inhibitors were pre-incubated with native sPLA2 from *Crotalus durissus terrificus* (20 μL, 2 mg/mL) in reaction buffer (Tris-HCl 50 mmol/L buffer pH 8.0, 2 mmol/L of calcium) for one minute prior to the addition of substrate at 37 °C, and two min prior to the addition of sPLA2 substrate (4 μmol/L). Enzymatic reactions were carried out at 37 °C for 30 min and absorbance at 405 nm was recorded at regular intervals of 10 min. In the graph, each point represents the mean and SD of six replicates, and X indicates compounds used and its respective concentration.

In the COX-1 and COX-2 cyclooxygenase inhibition assay, we used the COX activity assay kit (Cayman Chemical, item number 760151) to investigate the effect of 8CR on the enzymatic activity of COX enzymes and compare them with another COX inhibitor, DuP-697 (Cayman Chemical, item number 70645), and SC-560 (Cayman Chemical, item number 70340). DuP-697 is a member of the diaryl heterocyclic group of selective COX-2 inhibitors and SC-560 is a member of the diaryl heterocyclic class of cyclooxygenase (COX) inhibitors, which includes celecoxib (Celebrex™) and rofecoxib (Vioxx™). Each commercial inhibitor was dissolved following the manual instructions provided by the manufacturer, and 8CR was dissolved in DMSO. All of the concentrations of inhibitors were prepared and diluted in COX assay buffer and the concentrations were adjusted. These inhibitors were pre-incubated with the enzyme in reaction buffer for one minute prior to the addition of arachidonic acid (AA). Assays were performed using 100 units of ovine COX-1 or ovine COX-2 (one unit of enzyme consumes one nanomole of oxygen per minute at 37 °C) in 0.1 M/L Tris-HCl buffer, pH 8.0, containing 20 μM AA, 5 mmol/L EDTA, 2 mmol/L phenol. Assays were initiated by the addition of 20 μM AA; the colorimetric COX assay was measured by monitoring the appearance of colorimetric oxidized *N*,*N*,*N*′,*N*′-tetramethyl-*p*-phenylenediamine (TMPD) at 590 nm, and the COX activity was measured according to the manufacturer’s instructions.

### 4.5. Pharmacological Assay and Biochemical Assays

#### 4.5.1. Paw Edema

A paw edema assay was performed using previously described protocol [32]. Male Swiss mice (25 g) were anesthetized by inhaling halothane. Posterior paw edema was induced by a single subplantar injection of sPLA2. Paw volumes were measured immediately before the injection of the samples and at selected time intervals thereafter (0, 30, 60, 180, and 360 min) using a hydroplethysmometer (model 7150, Ugo Basile, Monvalle, Italy). The results are expressed as the increase in paw volume (mL) calculated by subtracting the initial volume. Each treatment was conducted for *n* = 5.

#### 4.5.2. Evaluation of Myonecrosis

The myotoxic activity was evaluated by the plasma creatine kinase (CK) measurement released from damaged muscle cells. For this, we used a commercial CK-NAc kit (Laborlab, London, UK), as described in [32]. The right gastrocnemius muscle was injected with 50 μL of 0.5 mg/mL sPLA2 sample, while the control mice received only an equal volume of 0.15 M NaCl. After 3 h, the animals were anesthetized and samples were collected from the abdominal cavity into tubes containing heparin as an anticoagulant. The plasma was stored at −10 °C for a maximum of 12 h before the assay. The level of CK was then determined with 40 μL of plasma, which was incubated for 3 min at 37 °C with 1.0 mL of the reagent according to the protocol kit. The resulting activity was expressed in U/L. Each treatment was conducted for *n* = 5.

#### 4.5.3. Determination of IL-1β, TNF-α, Prostaglandin E2 (PGE-2), and Malondialdehyde (MDA) Levels in Mice Paws

IL-1β, TNF-α, PGE-2, MDA, and COX-2 levels in mouse paw tissues were determined by colorimetric assay using tissue samples from paw tissue homogenization. Two hours after the injection of sPLA2, and two hours after the injection of cPLA2, five mice of each group were sacrificed, and the tissue samples were collected and weighed, snap frozen in liquid nitrogen, and stored at −80 °C to be processed for preparation of homogenates. Paw tissues (10% *w*/*v*) were homogenized in sodium phosphate buffer (0.1 M PBS, pH = 7.4) using a Disruptor Genie Cell Disruptor/Homogenizer (Scientific Industries, Inc.; 80 Orville Drive, Suite 102 Bohemia, New York 11716, USA). The homogenates were centrifuged at 9000× *g* for 20 min at 4 °C. The supernatants were collected, and IL-1β, TNF-α, PGE-2, and MDA levels were measured by respective enzyme-linked immunosorbent assay (ELISA) kits (Thermo Fisher Scientific, Waltham, MA, USA) according to the kit instructions. IL-1β, TNF-α, PGE-2, and MDA were determined using the following kits: IL-1β mouse ELISA kit (ab100704), mouse TNF-α ELISA kit (ab100747), prostaglandin E2 ELISA kit (ab133021) and lipid peroxidation (MDA) assay kit (colorimetric/fluorometric) (ab118970), mouse COX2 SimpleStep ELISA^®^ kit (ab210574), cytosolic phospholipase A2 assay kit (ab133090), and secretory phospholipase A2 assay kit (ab133089), respectively, and the protocol used for each biochemical analysis followed the manufacturer’s instructions (Abcam, Cambridge, MA, USA).

### 4.6. Statistical Analysis

The results are reported as the means ± SEM of the replicated experiments. The significance of the differences between the means was assessed by an analysis of variance followed by an analysis of variance (ANOVA) when several experimental groups were compared with the control group. The confidence limit for significance was 5%.

## 5. Conclusions

In conclusion, 8CR exhibited a generalized beneficial effect in terms of neutralizing the toxic effects of *Crotalus durissus terrificus* and sPLA2 snake venom, which included COX-2 neutralization in a dose-dependent manner. The inhibition of the mobilization of cytokines and other pro-inflammatory mediators generated by an increase in arachidonic acid metabolism contributed significantly to the decrease of free radical levels in cells. Thus, the systemic effects induced by the sPLA2 toxin from *Crotalus durissus terrificus* may be dependent on increased oxidative stress and the generation of reactive oxygen species. These effects are not completely neutralized by the action of commercial anti-venom, even by inhibiting the pro-inflammatory action of sPLA2 over the course of 60 min. In addition to inhibiting COX-2 activity, the compound 8CR also inhibited the phospholipase A2 activity and showed a strong antioxidant activity, and this set of qualities was essential for the 8CR compound to be more effective than the anti-venom or indomethacin against edema induced by sPLA2 from *Crotalus durissus terrificus*. In addition, the protein–protein interaction performed with sPLA2 as a molecular tool to evaluate the effect of 8CR treatment on the structure and function of sPLA2 strongly suggests that the compound did not substantially affect the protein structure as demonstrated by chromatographic data, as well as by the CD spectroscopy studies and tryptophan intrinsic fluorescence. The analysis of all of the results strongly supports the possible complementary anti-venom therapy for the treatment of snake bites, and a new class of polyvalent anti-inflammatory drugs.
